# Resveratrol prevents interleukin-1β-induced dysfunction of pancreatic β-cells^☆^

**DOI:** 10.1016/S1674-8301(10)60051-6

**Published:** 2010-09

**Authors:** Fang Chen, Xiaohua Zhou, Yan Lin, Changwen Jing, Tao Yang, Yong Ji, Yujie Sun, Xiao Han

**Affiliations:** a Key Laboratory of Human Functional Genomics of Jiangsu Province, Clinical Diabetes Centre of Jiangsu Province, Nanjing Medical University, Nanjing 210029, Jiangsu Province, China.; b Department of Endocrinology, Jiangsu Diabetes Center, the First Affiliated Hospital, Nanjing Medical University, Nanjing 210029, Jiangsu Province, China.; c Atherosclerosis Research Centre, Nanjing Medical University, Nanjing 210029, Jiangsu Province, China.

**Keywords:** resveratrol, interleukin-1β, peroxisome proliferator-activated receptor-γ, nitric oxide, nuclear factor-κB.

## Abstract

**Objective:**

Interleukin-1β (IL-1β) plays an important role in the development of type 1 and type 2 diabetes mellitus. Resveratrol, a polyphenol, is known to have a wide range of pharmacological properties *in vitro*. In this research, we examined the effects of resveratrol on IL-1β-induced β-cell dysfunction.

**Methods:**

We first evaluated the effect of resveratrol on nitric oxide (NO) formation in RINm5F cells stimulated with IL-1β using the Griess method. Next, we performed transient transfection and reporter assays to measure the transcriptional activity of peroxisome proliferator-activated receptor-γ (PPAR-γ). We also used Western blotting analysis to assess the effect of resveratrol on inducible nitric oxide synthase (iNOS) expression and nuclear factor-κB (NF-κB) translocation to the nuclei in cells treated with IL-1β. In addition, we assessed the transcriptional activity of NF-κB using an electrophoretic mobility shift assay (EMSA). Finally, we evaluated the effect of resveratrol on IL-1β–induced inhibition of glucose-stimulated insulin secretion in freshly isolated rat pancreatic islets.

**Results:**

Resveratrol significantly suppressed IL-1β-induced NO production, a finding that correlated well with reduced levels of iNOS mRNA and protein. The molecular mechanism by which resveratrol inhibited iNOS gene expression appeared to involve increased PPAR-γ activity, which resulted in the inhibition of NF-κB activation. Further analysis showed that resveratrol could prevent IL-1β-induced inhibition of glucose-stimulated insulin secretion in rat islets.

**Conclusion:**

In this study, we demonstrated that resveratrol could protect against pancreatic β-cell dysfunction caused by IL-1β.

## INTRODUCTION

Pancreatic β-cell dysfunction is the common characteristic of type 1 and type 2 diabetes mellitus. Although the initial events leading to the development of diabetes are not well characterized, inflammatory cytokines, including interleukin-1β (IL-1β), appear to play an important role in both types of diabetes[1]–[3]. IL-1β-induced dysfunction of the pancreatic islet β-cell is mainly due to the activation of nuclear factor-κB (NF-κB), which stimulates the expression of inducible nitric oxide synthase (iNOS) and the subsequent generation of nitric oxide (NO)[4]–[7]. It is well established that NO mediates cytokine-induced inhibition of insulin secretion in human islets[8]–[10].

Resveratrol is an edible polyphenolic phytoalexin present in different plant species, such as berries and peanuts. Resveratrol significantly improves mitochondrial function and protects against oxidative injury, so the body can resist numerous age-associated diseases, including cancer, Alzheimer's and cardiovascular disease[11],[16],[17]. Recently, resveratrol has received increasing attention for the prevention of type 2 diabetes because it not only improved insulin sensitivity of mice fed a high−fat diet but also increased insulin secretion in pancreatic β−cells[9],[12]. Furthermore, in streptozotocin−induced diabetic rats, resveratrol treatment significantly reduced blood glucose levels, and diabetic complications, such as renal and cardiac dysfunction, abated[13],[14]. Thus, resveratrol may be considered as an effective therapeutic agent for the treatment of diabetes mellitus.

Resveratrol has also been shown to increase PPAR -γ activity, and PPAR-γ activation protects pancreatic β-cells from IL-1β-induced cytotoxicity *via* the NF-kB pathway[20]–[23]. As a result, we chose to test the hypothesis that resveratrol could increase PPAR-γ activation and decrease NF-κB activation, which could then inhibit iNOS expression and NO formation induced by ILκB activation, which could then inhibit iNOS expression and NO formation induced by IL-1β.

## MATERIALS AND METHODS

### Reagents

Recombinant human IL-1β was purchased from R&D System, USA. Resveratrol was purchased from Sigma, USA. Anti-iNOS rabbit polyclonal antibody was purchased from Santa Cruz Biotechnology, USA. Horseradish peroxide-conjugated anti-mouse or anti-rabbit IgG were obtained from Amersham, USA. The PPAR-γ luciferase reporter construct was purchased from Clontech, USA. The Detergent Compatible (DC) Protein Assay kit was purchased from Bio-Rad USA. *Taq*Man one-step PCR kit and Assays-on-Demand gene expression products were from Applied Biosystems, USA. The luciferase assay system was obtained from Promega, USA.

### Cell culture

RINm5F, a rat insulinoma cell line, was obtained from ATCC, USA. The cells were grown in RPMI 1640 supplemented with 10% fetal bovine serum (FBS), 10 mmol/L HEPES, 2 mmol/L L-glutamine, 1 mmol/L sodium pyruvate, 100 U/mL penicillin, and 100 µg/mL streptomycin at 37°C in a humidified atmosphere containing 95% air and 5% CO_2_.

### Islet purification and culture

All animal studies were performed according to the guidelines established by the Research Animal Care Committee of Nanjing Medical University, China. Male Sprague-Dawley rats (200-250 g, purchased from Nanjing Medical University Laboratory Animal Centre, China) were used. Islet isolation and culture were performed as described previously[24]. Freshly isolated islets were transferred to sterile six-well dishes and cultured in RPMI 1640 containing 11.1 mmol/L glucose supplemented with 10% FBS, 10 mmol/L HEPES, 100 U/mL penicillin and 100 µg/mL streptomycin. The islets were allowed to equilibrate for 3 h, at which point they were counted and transferred into six well plates (300 islets per well for protein extraction) or 48 well plates (8 islets per well for glucose-stimulated insulin secretion) and cultured overnight at 37°C in 5%CO_2_-95% air. The islets were then pretreated with resveratrol (30 µmol/L) for 2 h and IL-1β (0.5 ng/mL) was added and the islets were further incubated for 24 h.

### Real-time reverse transcription PCR (real-time RT-PCR)

Total cellular RNA was extracted by TRIzol reagent (Invitrogen, USA) according to the manufacturer's protocol. After quantification, 1 µg total RNA was reverse-transcribed using the M-MLV reverse transcription system (Promega). cDNA aliquots corresponding to equal amounts of RNA were used for quantification of mRNA by real-time RT-PCR using the ABI Prism 7000 Sequence Detection System (Applied Biosystems). The reaction system contained cDNA, forward and reverse primers, and SYBR GREEN PCR Master Mix (Applied Biosystems). The *iNOS* gene primers were as follows: forward primer, 5′-CTCACTGTGGCTGTGGTCACCTA-3′ and reverse primer, 5′-GGGTCTTCGGGCTTCAGGTTA-3′. All data were analyzed using β-actin as an internal standard.

### Western blotting analysis

RINm5F cells were lysed in an ice-cold lysis buffer containing 50 mmol/L Tris-HCl (pH 7.4), 1% NP-40, 150 mmol/L NaCl, 1 mmol/L EDTA, 1 mmol/L phenylmethylsulfonyl fluoride, and complete proteinase inhibitor cocktail (one tablet per 10 mL; Roche, USA). Nuclear and cytoplasmic extracts were prepared using the nuclear extraction kit (Pierce, USA). After protein content determination using the DC Protein Assay kit, Western blotting was performed as described elsewhere[25].

### NO assay

RINm5F cells were cultured in 48-well dishes for 24 h, and pretreated with or without resveratrol for 2 h, and then incubated with IL-1β for 24 h. The medium was sampled for NO determination using the Griess method. Each experiment was performed in triplicate and repeated three times independently for reproducibility.

### Transient transfection and luciferase reporter assays

PPAR-γ transcriptional activity was assessed in RINm5F cells using the PPAR-γ luciferase reporter construct. We used a plasmid containing the β-galactosidase gene driven by the cytomegalovirus promoter (Clontech) as an internal control. RINm5F cells grown in 24-well dishes were transfected with the PPAR-γ luciferase reporter construct and β-galactosidase using the Lipofectamine Plus transfection kit according to the manufacturer's instructions (lnvitrogen). Twenty-four h after transfection, the cells were pretreated with resveratrol (30 µmol/L) for 2 h and followed with IL-1β (0.5 ng/mL) for 4 h. After the cells were lysed using 1×passive lysis buffer, luciferase activity was determined as previously described[26].

### Electrophoretic mobility shift assay (EMSA)

Nuclear extracts were prepared from RINm5F cells pretreated with resveratrol (30 µmol/L) for 2 h, with the addition of IL-1β (0.5 ng/mL) for 12 h by using the NE-PERTM Nuclear and Cytoplasmic Extraction Reagents (Pierce). The following oligonucleotide containing a consensus NF-κB binding site was used: 5′-AGTTGAGGGGACTTTCCCAGGC-3′. Oligonucleotides were end-labelled with the Biotin 3′-End DNA Labeling Kit (Pierce). Electrophoretic mobility shift assay (EMSA) was performed using a LightShift Chemiluminescent EMSA Kit (Pierce). Binding reactions were performed as follows: nuclear extracts (10 µg protein) and 1×binding buffer with 2.5% glycerol, 5 mmol/L MgCl_2_, 50 ng/µL poly (dI-dC), 0.05% NP-40, and 20 fmol biotin 3′-end labeled double-stranded oligonucleotides were incubated on ice for 20 min in a volume of 20 µL. DNA-protein complexes were resolved on non-denaturing 6% polyacrylamide gels at 100 V for 2 h. After gel electrophoresis, the DNA-protein complexes were transferred to a positively charged nylon membrane (Pharmacia, USA) and detected using chemiluminescence (Pierce).

### Glucose-stimulated insulin secretion assay

Isolated rat islets were seeded in 250 µL RPMI-1640 with 11.1 mmol/L glucose in 48-well dishes, and treated with appropriate drugs for 24 h as described above. Following preincubation for 1 h in Krebs-Ringer Bicarbonate (KRB) buffer containing 3.3 mmol/L glucose, the islets were treated for 1 h in KRB buffer and with low (3.3 mmol/L) or stimulatory (16.7 mmol/L) concentrations of glucose. The supernatants were then obtained and frozen at −70°C for subsequent determination of insulin concentration. The insulin levels were measured using RIA as described previously[27].

### MTT assays

Cell viability was determined using the MTT [3-(4, 5-dimethylthiazol-2-yl)-2,5-diphenyltetrazolium bromide] assays. Briefly, the cells were seeded in 96-well dishes at 1×10^4^ to 2×10^4^ cells per well, and pretreated with or without resveratrol for 2 h and then IL-1β was added for 24 h. Each well was then supplemented with 10 µL MTT (Sigma) and incubated for 4 h at 37°C. The medium was then removed, and 150 µL dimethyl sulfoxide (Sigma) were added to solubilize the MTT formazan. The optical density was read at 490 nm.

### Statistical analysis

Statistical analysis was performed with statistical analysis software (SPSS 11.0 software, SPSS Inc., USA). Comparisons between two groups were performed using Student's *t* tests, and among multiple groups by one-way ANOVA tests. Results were presented as mean±SEM. *P* < 0.05 was considered to have significant difference.

## RESULTS

### Resveratrol attenuated IL-1β-stimulated NO formation in pancreatic β-cells

**Fig. 1:**
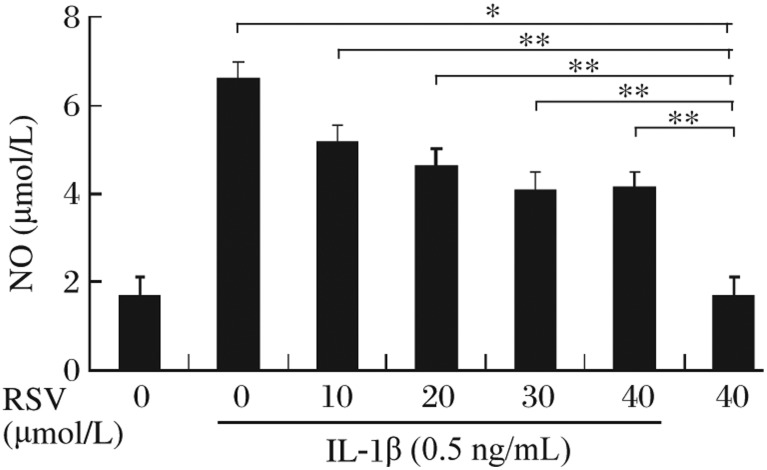
Resveratrol inhibited IL-1β-stimulated NO formation. IL-1β significantly induced NO formation compared with the control group (*P < 0.05), and resveratrol reversed this induction effect in a dose-dependent manner in RINm5F cells (**P < 0.01). RSV: resveratrol; NO: nitric oxide.

It has been reported that IL-1β-mediated destruction of pancreatic β-cells is caused by an increased production of NO[8]. We found that RINm5F cells in a normal state released 1.68±0.09 µmol/L of NO, whereas cells treated with IL-1β exhibited markedly increased NO production. No significant difference of NO production was found between control RINm5F cells and cells treated with resveratrol (40 µmol/L) alone. However, resveratrol significantly inhibited IL-1β-induced NO production in a dose-dependent manner, with the maximum inhibition occurring at the concentration of 30 µmol/L resveratrol (***Fig.1***).

### Resveratrol inhibited IL-1β-stimulated mRNA and protein expression of iNOS

**Fig. 2:**
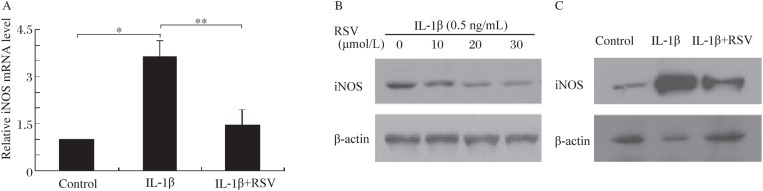
Resveratrol inhibited IL-1β-stimulated iNOS mRNA and protein expression. A: IL-1β significantly induced iNOS mRNA expression (*P < 0.05), and resveratrol inhibited this effect (**P < 0.01). B: IL-1β induced iNOS protein expression in RINm5F cells; C: Resveratrol significantly inhibited IL-1β-induced iNOS protein expression in rat islets. RSV: resveratrol; iNOS: inducible nitric oxide synthase; IL-1β: interleukin-1β.

iNOS expression plays a critical role in NO production[28]. In order to determine whether resveratrol inhibited NO production by suppressiing iNOS expression, we investigated the mRNA and protein levels of iNOS by real-time RT-PCR and Western blotting, respectively. As shown in ***Fig. 2A***, iNOS transcription was markedly increased when cells were treated with IL-1β. Resveratrol attenuated IL-1β-induced changes of iNOS mRNA. Moreover, resveratrol inhibited IL-1β-induced iNOS protein expression in RINm5F cells (***Fig. 2B***) and in rat islets (***Fig. 2C***). Therefore, resveratrol mediated a significant inhibition of iNOS expression.

### Resveratrol inhibited the effect of IL-1β on PPAR-γ activity in RINm5F cells

**Fig. 3:**
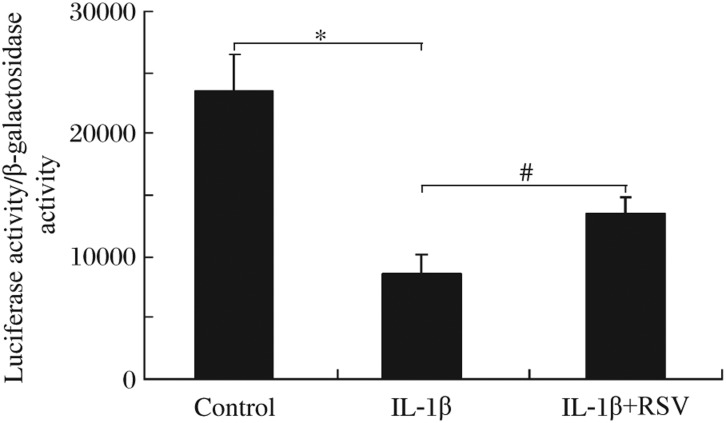
Resveratrol attenuated IL-1β-induced change of PPAR-γ activity. IL-1β significantly decreased the transcriptional activity of PPAR-γ (*P < 0.05) and resveratrol could inhibit this effect (^#^*P* < 0.05). RSV: resveratrol; IL-1β: interleukin-1β.

To determine whether the regulatory function of resveratrol was mediated by PPAR-γ, we investigated the effect of this compound on IL-1β-induced PPAR-γ transcriptional activity by using a PPAR-γ luciferase reporter construct. When RINm5F cells were stimulated with IL-1β, PPAR-γ transcriptional activity was significantly decreased; however, resveratrol attenuated this IL-1β-induced change of PPAR-γ activity (***Fig. 3***).

### Resveratrol inhibited IL-1β-induced activation of the NF-κB pathway

**Fig. 4:**
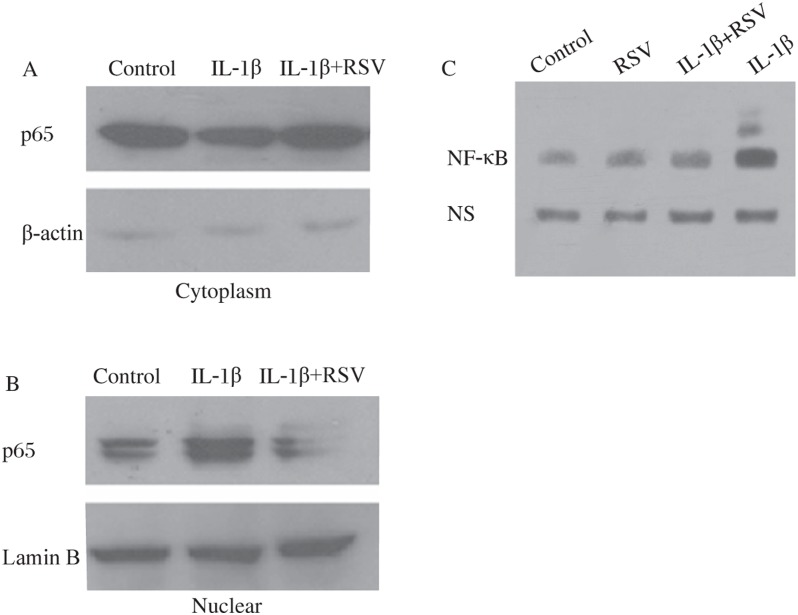
Resveratrol reversed NF-κB translocation from the cytoplasm to the nuclei induced by IL-1β. A: Protein level of cytoplasmic NF-κB (p65) was decreased in RINm5F cells treated with IL-1β (0.5 ng/mL), and resveratrol reversed the effects remarkably. B: Protein level of p65 in the nucleus was increased in RINm5F cells treated with IL-1β (0.5 ng/mL), and the increase was reversed by resveratrol. C: protein levels of NF-κB in different groups. RSV: resveratrol; IL-1β: interleukin-1β; NS: Non-specific.

NF-κB is implicated in the transcriptional regulation of cytokine-induced iNOS expression. Therefore, we investigated whether resveratrol could reverse the activation of NF-κB induced by IL-1β. When RINm5F cells were stimulated with IL-1β (0.5 ng/mL), NF-κB (p65) levels in the cytoplasm decreased while NF-κB levels in the nucleus increased. As shown in ***Fig. 4A***, resveratrol inhibited IL-1β-induced NF-κB translocation to the nuclei. Furthermore, the transcriptional activity of NF-κB was decreased by resveratrol as demonstrated by EMSA (***Fig. 4B***).

### Resveratrol improved glucose-stimulated insulin secretion in rat islets treated with IL-1β

**Fig. 5:**
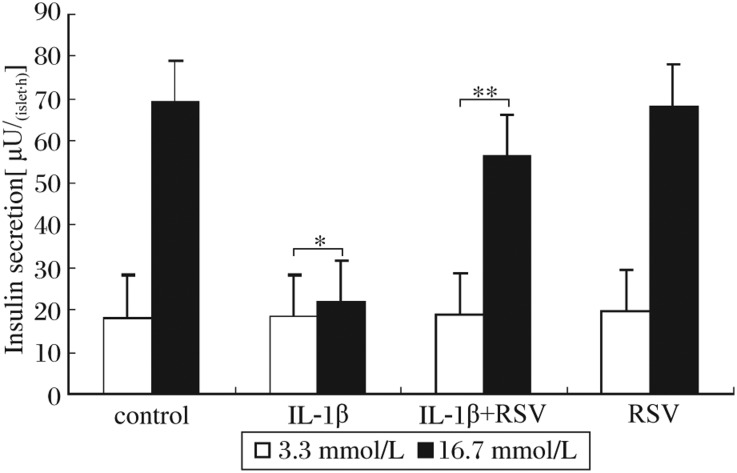
Resveratrol improved glucose-stimulated insulin secretion of rat islet under treatment with IL-1β. IL-1β significantly inhibited insulin secretion stimulated with high glucose (*P < 0.05) and resveratrol could improve the function of islets treated with IL-1β (**P < 0.01). RSV: resveratrol; IL-1β: interleukin-1β.

We assayed glucose-stimulated insulin secretion using rat pancreatic islets isolated from male Sprague-Dawley rats to augment the results observed in the cell line studies (***Fig. 5***). After exposure to IL-1β for 24 h, the islets showed significantly decreased insulin secretion in response to stimulation by 16.7 mmol/L glucose. Pretreatment with resveratrol (30 µmol/L) blocked IL-1β-induced reduction in insulin levels and restored islet insulin secretion to near control levels. Resveratrol alone did not affect insulin release compared to controls.

### Resveratrol and IL-1β had no effect on pancreatic β-cell viability

NO production has been implicated in IL-1β-induced pancreatic β-cell dysfunction and disruption[8],[29]. As resveratrol inhibited IL-1β-induced NO production, we evaluated the potential effects of resveratrol and IL-1β on pancreatic β-cell viability using the MTT assays. As shown in ***Table 1***, treatment with IL-1β (0.5 ng/mL) or resveratrol (30 µmol/L) alone or in combination did not significantly inhibit the viability of RINm5F cells. These observations suggested that resveratrol alleviated the dysfunction of pancreatic β-cell induced by IL-1β without affecting cell survival.

**Table 1:** Resveratrol and interleukin-lβ (IL-lβ) had no effect on pancreatic β-cell viability

	Group 1	Group 2	Group 3
Control	0.57 ± 0.01	0.58 ± 0.02	0.56 ± 0.01
IL-lβ	0.56 ± 0.02	0.58 ± 0.02	0.55 ± 0.04
RSV	0.57 ± 0.02	0.58 ± 0.02	0.57 ± 0.01
IL-lβ+ RSV	0.56 ± 0.02	0.58 ± 0.02	0.56 ± 0.02

Group 1, 2 and 3 were represntative of three independent experiments, respectively.

RSV: resveratrol; IL-lβ: interleukin-lβ.

(*n =* 6)

## DISCUSSION

Type 2 diabetes mellitus is a chronic metabolic syndrome caused by insulin deficiency. The underlying mechanisms are not fully understood, but it is apparent that inadequate functional β-cells are important. The proinflammatory cytokine, IL-1β, plays a crucial role in negatively regulating β-cell function in type 2 diabetes mellitus[1],[2]. Resveratrol, an edible polyphenolic phytoalexin, has been shown to prevent IL-1β-mediated destruction in several cell types[30],[31]. In this study, we have evaluated the protective effect of resveratrol on IL-1β-induced dysfunction in pancreatic β-cells. The results showed that resveratrol induced a significant reduction in NO production, which correlated well with reduced levels of iNOS mRNA and protein in RINm5F cells treated with IL-1β. Furthermore, resveratrol significantly enhanced PPAR-γ activity and attenuated NF-κB activation in IL-1β-treated cells. Most importantly, resveratrol could improve glucose-stimulated insulin secretion in IL-1β-treated rat islets.

It is well known that IL-1β-induced dysfunction of islet β-cells is mainly due to the generation of NO[28]. Interestingly, some reports showed that resveratrol contributes to its protective effect through decreasing the release of NO[32]–[34]. Thus, we postulated that resveratrol could prevent production of NO to protect β-cells. Our results indeed provided evidence that resveratrol could reduce IL-1β-induced NO formation. This finding suggests that inhibition by resveratrol of the level of NO induced by IL-1β in RINm5F cells may be one of its mechanisms of action. On the other hand, the inhibition of NO production by resveratrol could be due to the inhibition of iNOS expression. To test this possibility, we analyzed the expression level of iNOS in resveratrol-treated cells. We found that resveratrol could significantly inhibit IL-1β-induced iNOS mRNA and protein expression, indicating that resveratrol regulated NO production through iNOS expression at the level of gene transcription. To ensure that the cell line did not bias our results, we evaluated the effect of resveratrol on IL-1β-stimulated iNOS protein expression in rat islets and obtained the same results as the RINm5F cells.

PPAR-γ is a nuclear receptor that plays a crucial role in the protection of pancreatic β-cells[23],[25]. In this paper, we found that resveratrol could increase transcriptional activity of PPAR-γ in pancreatic β-cells treated with IL-1β. It has been reported that PPAR-γ could inhibit transcriptional activity of NF-κB, which plays an important role in IL-1β-induced β-cell dysfunction and death[22],[36]–[38]. Based on our results, we hypothesized that resveratrol exerted its protective actions through enhancement of PPAR-γ transcriptional activity, which resulted in decrease of NF-κB activity, reducing the production of NO and improving the function of pancreatic β-cells treated with IL-1β.

It should also be mentioned that 0.5 ng/mL IL-1β could significantly inhibit rat islet glucose-stimulated insulin secretion, while this concentration of IL-1β did not significantly affect RINm5F cell viability over 24 h. Additionally, resveratrol alone (30 µmol/L) did not reduce RINm5F cell viability, but it could significantly improve glucose-stimulated insulin secretion of rat islets treated with IL-1β, which suggested resveratrol exerted its beneficial effects on pancreatic β-cells without having adverse side effect.

In summary, we have demonstrated a profound inhibitory effect of resveratrol on IL-1β-induced dysfunction of β-cells using an insulinoma cell line and isolated rat pancreatic islets. The results of this study will provide valuable information for the development of drugs designed to combat type 2 diabetes.
